# Mutant ubiquitin reduces Aβ plaques

**DOI:** 10.18632/aging.101598

**Published:** 2018-10-15

**Authors:** Bert M. Verheijen, Fred W. van Leeuwen

**Affiliations:** 1 Department of Neuroscience, Maastricht University, Maastricht, The Netherlands

**Keywords:** mutant ubiquitin, ubiquitin-proteasome system, amyloid β, Aβ plaques, γ-secretase, proteostasis, Alzheimer’s disease

**Figure 1:**
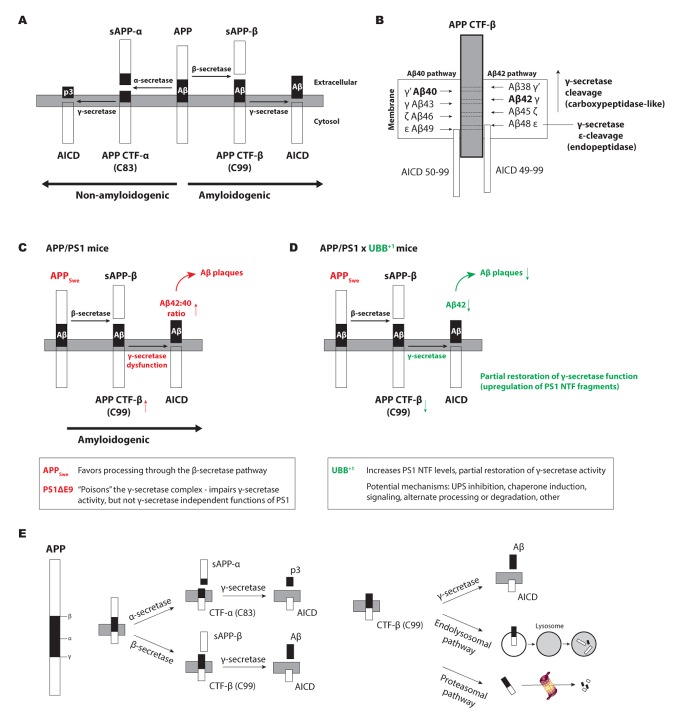
**Mutant ubiquitin (UBB^+1^) reduces Aβ plaques in APPPS1 mice.** (**A**) Schematic diagram of amyloid precursor protein (APP) processing leading to Aβ. (**B**) γ-secretase is a multimeric enzyme complex that cleaves APP C-terminal fragment (CTF) to produce Aβ and an APP intracellular domain (AICD). (**C**) Transgenic APPPS1 mice carry two mutations that represent early-onset AD and develop age-related amyloid plaque pathology. (**D**) In APPPS1xUBB+1 mice, APP CTF-β levels are reduced and γ-secretase function is partially restored. (**E**) Besides secretases, alternate protein degradation pathways have been reported to degrade APP and Aβ.

Deposition of extracellular amyloid plaques is one of the main pathological features of Alzheimer’s disease (AD), the most common cause of dementia. These plaques are composed primarily of aggregated amyloid β-peptide (Aβ), which is generated through proteolytic processing of the amyloid precursor protein (APP) by β- and γ-secretases (Figure 1A, B). Genetic mutations associated with dominant hereditary dementia, i.e., *APP*, *PSEN,* and *PSEN2* mutations, strongly suggest that alterations in Aβ are sufficient to induce neurodegenerative disease. According to the “amyloid hypothesis”, accumulation of Aβ in brain is the primary influence driving AD pathogenesis [1]. Therefore, lowering Aβ is a major therapeutic goal in AD. This might be achieved by controlling the production, aggregation or clearance of Aβ.

The ubiquitin-proteasome system (UPS) is a highly regulated mechanism for protein breakdown in cells. It has been put forward that impaired UPS-mediated proteolysis contributes to AD pathogenesis, but the significance of the UPS in Aβ metabolism remains largely unclear. To study the effects of a chronically impaired UPS on Aβ pathology *in vivo*, we crossed APPPS1 mice with transgenic mice expressing mutant ubiquitin (UBB^+1^), a protein-based UPS inhibitor [2,3]. APPPS1 mice express a chimeric mouse/human mutant APP (Mo/HuAPP695swe-K595N/M596L) and a mutant human presenilin 1 (PS1-ΔE9), mutations that both represent early-onset AD, in CNS neurons and develop β-amyloid deposits in brain (Figure 1C). Unexpectedly, the APPPS1xUBB^+1^ crossbred mice showed a decrease in plaques during aging [4]. Also, levels of soluble Aβ42 were reduced in brain, suggesting that lower levels of Aβ42 might contribute to the decreased plaque load.

To investigate the effects of UBB^+1^ expression on APP processing, we carried out secretase activity measurements on brain tissue samples from different mouse lines [5]. In APPPS1 mice, a partial decrease in γ-secretase activity was found compared to wild-type mice, in agreement with disruption of normal γ-secretase function by the PS1-ΔE9 mutation present in these animals (presenilin is the catalytic component of the γ-secretase complex). Interestingly, in APPPS1xUBB^+1^ triple transgenic mice, γ-secretase activity was partially restored, specifically at 6 months of age (Figure 1D). Onset of amyloid plaque pathology in the APPPS1 mouse model occurs at approximately the same age. To measure γ-secretase activity, an internally quenched fluorogenic peptide substrate containing APP C-terminal fragment (CTF)-β was used: proteolysis at Aβ40-, Aβ42-, and Aβ43-generating cleavage sites in the substrate resulted in enhanced fluorescence. Therefore, changes in γ-secretase activity indicated altered carboxypeptidase-like cleavage in this assay. How UBB^+1^ exerts this stimulating effect on γ-secretase is not clear, but a potential mechanism may involve regulation of presenilin expression [5].

In addition to the partial recovery of γ-secretase activity, protein levels of APP CTF-β (C99), as determined in immunoblots, were reduced in triple transgenes compared to APPPS1 mice (Figure 1D) [5]. C99 is the amyloidogenic substrate for γ-secretase, whose intraneuronal accumulation has also been suggested to be toxic. Besides increased processing by γ-secretase that lowers C99 levels, it is possible that alternative degradation of APP CTFs plays a role in the UBB^+1^-induced reduction in Aβ load. For example, compensatory turnover of CTF-β fragments in brain could occur via the endolysosomal pathway or through other proteasome-dependent pathways (Figure 1E). Moreover, UBB^+1^ can increase chaperone expression, potentially assisting in degradation of Aβ [6]. Cellular effects of UBB^+1^ expression are more complex than previously appreciated and should be investigated in more detail in future studies. Non-canonical APP processing routes (e.g., δ-secretase, η-secretase (MT5-MMP), Meprin-β, and caspase cleavage) were not examined in our experiments.

The reported findings support a role of the UPS proteolytic pathway in the accumulation of amyloid peptide. Additionally, the findings may have important translational implications: γ-secretase inhibitors have been proposed as a therapeutic option in AD, but clinical trials have indicated that these are associated with adverse effects and can actually worsen disease outcome in patients. Accumulation of γ-secretase substrates potentially contributes to cognitive worsening. We suggest that recovery of γ-secretase function, e.g., via stimulation of carboxypeptidase function, should be explored as a therapy to lower Aβ in AD. We did not find an ameliorating effect of UBB^+1^ expression on behavioral deficits in APPPS1 mice [5], but it should be noted that UBB^+1^ induces behavioral impairments itself [3,7], which could mask a beneficial effect of reducing Aβ.

Importantly, AD is a multifactorial disease that is not just characterized by accumulation of Aβ, but also involves other pathological changes, e.g., accumulation of hyperphosphorylated tau protein in neurofibrillary tangles and alterations in synapses and glial cells, at different time points during the disease course [8]. While the contribution of γ-secretase dysfunction and Aβ in rare familial forms of AD is well established, it remains unclear whether Aβ is a key initiating factor in sporadic cases or merely coincides with the true pathogenic entities. Few sporadic AD patient samples display qualitative changes in γ-secretase function, for example. Aβ may be necessary, but not sufficient, to cause AD. The normal functions of APP and its proteolytic fragments remain poorly understood, with proposed functions ranging from transcriptional regulation and neurodevelopment to synaptic functions and anti-microbial properties. Finding out how APP and its metabolites, and other factors implicated in AD pathology (tau, glial cells), interact with specific cellular mechanisms, like the UPS and additional proteolytic pathways, will undoubtedly provide new insights into AD pathogenesis and may result in novel therapeutic strategies. In view of therapy, approaches targeting Aβ may fail once disease processes become independent of Aβ. Therefore, diagnostic testing for early alterations in Aβ may be essential.
